# Multi-source financing for tuberculosis treatment in China: key issues and challenges

**DOI:** 10.1186/s40249-021-00809-4

**Published:** 2021-03-10

**Authors:** Qian Long, Wei-Xi Jiang, Hui Zhang, Jun Cheng, Sheng-Lan Tang, Wei-Bing Wang

**Affiliations:** 1 Global Health Research Center, Duke Kunshan University, Jiangsu, China; 2 National Center for Tuberculosis Control and Prevention, China CDC, Beijing, China; 3 Duke Global Health Institute, Duke University, Durham, NC USA; 4 Department of Epidemiology, School of Public Health & Key Laboratory of Public Health Safety (Ministry of Education), Fudan University, 138 Yi Xue Yuan Road, Shanghai, 200032 China

**Keywords:** Tuberculosis, Medical cost, Financial protection, Universal health coverage, Multi-sector engagement, China

## Abstract

**Background:**

The End Tuberculosis (TB) Strategy of the World Health Organization highlights the need for patient-centered care and social protection measures that alleviate the financial hardships faced by many TB patients. In China, TB treatments are paid for by earmarked government funds, social health insurance, medical assistance for the poor, and out-of-pocket payments from patients. As part of Phase III of the China-Gates TB project, this paper introduces multi-source financing of TB treatment in the three provinces of China and analyzes the challenges of moving towards universal coverage and its implications of multi-sectoral engagement for TB care.

**Main text:**

The new financing policies for TB treatment in the three provinces include increased reimbursement for TB outpatient care, linkage of TB treatment with local poverty alleviation programs, and use of local government funds to cover some costs to reduce out-of-pocket expenses. However, there are several challenges in reducing the financial burdens faced by TB patients. First, medical costs must be contained by reducing the profit-maximizing behaviors of hospitals. Second, treatment for TB and multi-drug resistant TB (MDR-TB) is only available at county hospitals and city or provincial hospitals, respectively, and these hospitals have low reimbursement rates and high co-payments. Third, many patients with TB and MDR-TB are at the edge of poverty, and therefore ineligible for medical assistance, which targets extremely poor individuals. In addition, the local governments of less developed provinces often face fiscal difficulties, making it challenging to use of local government funds to provide financial support for TB patients. We suggest that stakeholders at multiple sectors should engage in transparent and responsive communications, coordinate policy developments, and integrate resources to improve the integration of social protection schemes.

**Conclusions:**

The Chinese government is examining the establishment of multi-source financing for TB treatment by mobilization of funds from the government and social protection schemes. These efforts require strengthening the cooperation of multiple sectors and improving the accountability of different government agencies. All key stakeholders must take concrete actions in the near future to assure significant progress toward the goal of alleviating the financial burden faced by TB and MDR-TB patients.

**Graphic abstract:**

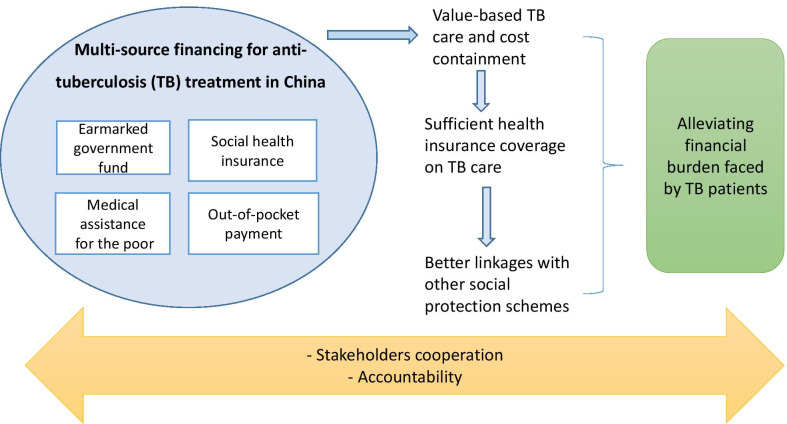

## Background

Tuberculosis (TB) continues to be a major global health challenge and was responsible for 1.4 million deaths worldwide during 2019 [1]. Among infectious diseases, TB is a leading cause of death and it disproportionately afflicts poor and socially marginalized populations, especially in Asia and Africa. Limited access and adherence to treatment, which are mainly due to insufficient financial support, are the major barriers to TB control in many low- and middle-income countries. A systematic review in 2014 that assessed total costs (direct medical costs, direct non-medical costs, and income loss) faced by TB patients from low- and middle-income countries found that this total accounted for more than half of annual individual income and about 40% of annual household income [2]. The Western-Pacific Region of the World Health Organization (WHO) has 17% of the global TB burden. Several countries in this region conducted national surveys of TB costs and found that 35% to 70% of TB patients faced catastrophic costs due to their need for medical care [3].

In 2014, the 67th World Health Assembly endorsed the End TB Strategy, whose goals were to reduce the TB incidence rate by 90% and the number of TB deaths by 95% by 2035 (relative to 2015) and to assure that "no TB-affected families were facing catastrophic costs due to TB" by 2020 [4]. This strategy highlighted the global progress towards universal health coverage (UHC), defined as universal access to high-quality health care without causing financial hardship as fundamental to "ending the global TB epidemic" [4, 5]. Achieving these goals will require bold national policies, increased funding, and support from within and outside the healthcare sector. Many low- and middle-income countries are exploring new programs to improve access to care and alleviate the financial burdens faced by patients with TB, such as free essential TB testing, free access to first-line anti-TB medicines, improved enrollment of health insurance, health insurance reforms, and compensation through other social protection schemes [6]. However, more progress is needed before these countries can provide universal coverage for TB patients.

China is among the top three countries in the world in terms of TB burden, and had 833,000 new cases of TB and 65,000 new cases of drug-resistant TB (DR-TB) during 2019 [1]. Payments for TB treatment in China are from the government, social funds, and patients themselves. Under the national TB program, earmarked funds from the central government pay for first-line anti-TB drugs and essential TB tests, including sputum smear tests and chest X-ray examinations. Social health insurance also covers part of the medical costs for TB treatment. Since the early 2000s, the Chinese government has supported and gradually expanded coverage of three basic medical insurance schemes: the Urban Employee Basic Medical Insurance (UEBMI), the Urban Residents Basic Medical Insurance (URBMI), and the rural New Cooperative Medical Scheme (NCMS). In recent years, URBMI was integrated with NCMS in some provinces and renamed as the Urban and Rural Residents Basic Medical Insurance (URRBMI). Overall, UEBMI provides better coverage for outpatient and inpatient care than URRBMI; the latter generally has limited coverage for outpatient care and a lower reimbursement rate for inpatient care at high-level hospitals. TB patients must pay a deductible, a co-payment, and fees for medications and examinations that are not covered by government funds or health insurance. However, poor TB patients can apply for medical assistance from the Civil Affairs Agency, which often covers part of the medical costs for treatment after health insurance reimbursement. Although earmarked government funds and basic medical insurance cover essential TB services, ancillary drugs and examinations are billed separately, and out-of-pocket payments can be high. Many TB patients, particularly those with multi-drug resistant TB (MDR-TB), face catastrophic expenditures [7, 8].

In 2016, the Chinese government issued the Plan of Healthy China 2030 in response to Sustainable Development Goals (SDGs) of the United Nations, and this included a goal of continuing to decrease the TB burden in China. In cooperation with the Bill & Melinda Gates Foundation, the Chinese Center for Disease Control and Prevention (CDC) is leading the promotion of a comprehensive TB control model that includes mobilization of multi-source financing for TB care. This paper introduces the new financing policy for TB treatment in the three provinces of China aiming to reduce the financial burden faced by TB patients, and analyzes the challenges of moving towards universal coverage for TB care and its implications of multi-sector engagement for TB control.

## Main text

### Context

Phase III of the China-Gates TB program was implemented in three provinces: Zhejiang, Jilin, and Ningxia (Table 1). In 2018, Zhejiang had a per capita gross domestic product (GDP) that was 1.5-times above the national average, whereas Jilin and Ningxia had per capita GDPs below the national average. The TB incidence rates in all three provinces have continued to decrease over time, and were lower than the national average during recent years.

**Table 1:** Tuberculosis incidence rates and health expenditures in the three project provinces and nationwide

Province	Population (× 10 000), 2018	GDP per capita (CNY), 2018	TB incidence rate/100 000, 2018	Total health expenditures (%), 2017			Urban medical expenditures/living expenditures (%), 2017	Rural medical expenditures/living expenditures (%), 2017
				Government	Social	Out-of-pocket		
Zhejiang	5737	98 643	45.3	21.2	51.8	27.0	5.9	7.6
Jilin	2704	55 611	47.4	28.2	39.0	32.8	10.8	13.6
Ningxia	688	54 094	36.7	33.5	38.5	28.0	9.6	11.3
Nationwide	139 538	64 644	59.3	28.9	42.3	28.8	7.3	9.7

Data source: 2019 China Health Statistical Yearbook

The National Health Account of China classified three sources of financing for healthcare: government expenditures, social expenditures (mostly health insurance payments and a smaller amount from donations by non-governmental organizations), and individual out-of-pocket payments [9]. Compared to the national averages, Ningxia had more government health expenditures, Zhejiang had more social health expenditures, and Jilin had more out-of-pocket expenditures. Medical expenditures as a percentage of total living expenses in urban and rural areas in Zhejiang were lower than the national average, but this percentage was above the national average in Jilin and Ningxia. In the three provinces, the percentage of reimbursement for inpatient care was 80% to 90% (Urban Employee Basic Medical Insurance, UEBMI) and 65% to 75% (Urban and Rural Residents Basic Medical Insurance, URRBMI /New Cooperative Medical Scheme, NCMS) during 2016. The coverage provided for outpatient care was generally more limited, particularly that from URRBMI/NCMS.

### New financing policies for anti-TB treatment in the three provinces

One of the main interventions in Phase III of the China-Gates TB project is to establish a mechanism for multi-source financing of anti-TB treatment by mobilizing social and government resources. This mobilization includes improving medical insurance coverage and contributions from local governments so that patients with TB and MDR-TB receive adequate financial protection. This project had a top-down approach, and interventions were implemented at all levels of each province. Compared to other interventions that strengthen TB services delivery, the present intervention took more time to achieve a consensus regarding new financing policies for TB treatments in the three provinces.

The new policies for financing TB treatment differed in the three provinces (Table 2). In Zhejiang, the strategy was to increase coverage for outpatient care provided by URRBMI. Thus, in May 2019, TB outpatient care was included in the benefits package for chronic diseases. This required that the percentage of outpatient reimbursement at primary health facilities was 70% or more. This provincial health insurance policy also allowed each city or county to decide on the reimbursement percentage for care of TB outpatients at secondary and tertiary hospitals, depending on the affordability of the local health insurance.

**Table 2:** New financing policies for tuberculosis (TB) treatments in the three project provinces

Province	Time of issue	Strategy	Coverage
Zhejiang	June 17, 2019	Improvement of outpatient care coverage in Urban and Rural Residence Basic Medical Insurance	TB included in the outpatient scheme for chronic diseases Outpatient reimbursement at primary health facility: ≥ 70%, At secondary and tertiary hospitals: dependence of affordability of health insurance funds in each city and county
Jilin	August 10, 2018	Linkage with rural poverty alleviation program and target poor rural patients with TB	Social and government funds: The medical costs should be shared by New Cooperative Medical Scheme, the medical scheme for severe diseases, and Medical Assistance and local government earmarked funds Out-of-pocket payment: Inpatient care: 10% Outpatient care: 20%
Ningxia	October 20, 2017	Improvement of outpatient care coverage in Urban and Rural Residence Basic Medical Insurance and Urban Employee Basic Medical Insurance	TB included in outpatient scheme for severe diseases in basic medical insurance schemes
	March 15, 2018	Contribution of local government in sharing medical costs for TB	Social and government funds: Medical costs should be shared by the basic medical insurance schemes and local earmarked government funds Out-of-pocket payment: Drug-resistant TB patients: 10% Drug-susceptible TB patients: 30%

Data source: Policy paper of the project provinces, 2019 June 30

In Jilin, the strategy was to mitigate the financial burden faced by poor TB patients from rural areas by providing them coverage from social and government funds. This policy was linked with a rural poverty alleviation policy that targeted poor TB patients from rural areas. Since August 2018, the medical costs of TB treatment were shared by NCMS, medical insurance for severe diseases, and a medical assistance scheme at the local civil affairs agency. If the financial support from the social protection schemes was insufficient, earmarked local government funds covered part of the costs. The goal was for poor rural TB patients to pay 10% of inpatient costs and 20% of outpatient costs out-of-pocket.

In Ningxia, the basic medical insurance schemes expanded the benefits package provided for care of TB outpatients in October 2017, and the local government committed earmarked funds to cover part of medical costs after reimbursements from medical insurance schemes in March 2018. Patients with DR-TB were required to pay 10% of the total treatment costs out-of-pocket, and patients with drug-susceptible TB were required to pay 30% of the total treatment costs out-of-pocket.

### Key issues and challenges of multi-source financing for anti-TB treatment in China

Is this financial protection sufficient for patients with TB and MDR-TB? The new financing policy in all three provinces began late in the project period. Thus, most TB and MDR-TB patients who received treatment 6 to 8 months before the project's final evaluation (June 2019) did not benefit from the new policy. Given the broader policy environment, and in the context of socio-economic development, several major challenges prevent moving towards universal coverage for the care of TB patients without suffering from financial hardships.

The first and greatest challenge is the reform of the financing of public hospitals in the Chinese health system. Since the 2000s, China started to transform the vertical TB control program led by the CDC into horizontally integrated TB care delivery model—the “TB designated hospital model”. TB designated hospitals are typically specialized infectious diseases hospitals or are general hospitals that have TB departments which focus on the diagnosis and treatment of TB and MDR-TB. Although earmarked funds from the central government cover first-line anti-TB drugs and essential examinations, over-prescription of second-line anti-TB drugs, expensive examinations, inpatient care, and prolonged treatment courses are common. This is partly because of doctors' knowledge and awareness of standard anti-TB treatments, and partly because of the profit-seeking behaviors of hospitals. The local health authority often asks the CDC to monitor the TB care provided by designated hospitals. However, the CDC does not have the authority to regulate hospital practices. Thus, efforts are needed to reverse the excessive growth of health expenditures and to remove perverse financial incentives in public health facilities.

A second challenge is that current health insurance policies do not accommodate the TB service delivery model in China. Drug-susceptible TB treatment is provided by county or city hospitals, and DR-TB treatment is provided by high-level hospitals. High-level hospitals have greater deductibles and reduced reimbursement relative to low-level facilities. In Zhejiang, the new policy required the percentage reimbursement for outpatient TB care at primary health facilities to be 70% or more; however, TB patients may not benefit due to inconsistencies in the TB service delivery model. The basic principal of health insurance is to the share financial risk among many individuals who need health care. Thus, health insurance agencies are unlikely to provide extensive coverage, such as exemption of deductibles or reduced co-payments, for a single disease, such as TB and MDR-TB. In addition, government investments in basic medical insurance schemes have increased steadily since 2009, and these medical insurance schemes are expected to cover increased health services. As a major source of paying for health services, health insurance agencies have limited capacity or incentive to monitor the profit-maximizing behaviors of healthcare providers, although this is necessary for containing medical costs. When total health expenditures increase, health insurance agencies struggle to maintain their funds, and face the dilemma of prioritizing health services by considering local disease burdens. These problems occurred during the negotiations used to develop new financing policies for TB treatment in the three provinces.

A third challenge is that the medical assistance program in the civil affairs agency only targets extremely poor populations. In fact, many patients with TB and MDR-TB face difficulties in paying for their care, but are ineligible for medical assistance. In addition, some patients who did qualify complained about the complex application procedures and the long time it takes to receive the subsidy. In Jilin, as an integral component of the rural poverty alleviation program, medical costs for TB treatment are directly deducted from TB designated hospitals, and eligible patients only pay 10% to 20% of total medical costs out-of-pocket. However, the financial burden currently faced by poor rural patients with TB requires further examination.

Another consideration is that the local governments in less developed provinces often face fiscal difficulties, and therefore invest less in social protection schemes. The local governments of Jilin and Ningxia committed funds to pay for part of the medical costs of TB treatment as a supplement to the social protection schemes to reduce out-of-pocket payments. However, these two provinces have lagging economic growth and limited government revenues. Moreover, it is challenging for the local CDC to negotiate and coordinate new financing policies for TB treatment among multiple sectors (Health Insurance Agency, Civil Affairs Bureau, and Financing Bureau) because they lack administrative authority and the stakeholders have different interests. This caused delays in issuing and implementing the new financing policies during the project period. The economic and health crisis caused by the COVID-19 pandemic also poses significant challenges for fundraising and mobilization of resources, particularly in less developed areas [10]. There is thus a need to monitor the sustainability of relying on government compensation to cover part of the medical costs of TB treatment in low resource settings, and the scale-up of such strategies should proceed with caution.

### Policy implications

One of the goals of the nation-wide Healthy China 2030 Plan is to reduce the percentage of personal health expenditures to less than 25% of all health expenditures by 2030 [9]. Alleviation of the financial hardships experienced by TB patients is in line with progress towards UHC. We learned from the implementation of multi-source financing for TB treatment that engagement and coordination of multiple sectors is vitally important, and believe that this approach can be extended to achieve sustainable and scalable health financing policies in general.

We recommend three general approaches to achieve these goals. First, stakeholders from multiple sectors should be engaged in transparent and responsive communications, and there should be a strong government commitment and leadership to address the challenges related to finances, policy development, and implementation. Quality of care, cost containment, and the effective use of limited resources are the major concerns of stakeholders. The Chinese government has tested and tried to scale-up public hospital reform to reverse the trend of increasing health expenditures. In 2019, the National Healthcare Security Administration (which is in charge of social health insurance schemes) introduced a pilot program—the Diagnosis Related Groups (DRGs) payment model. In line with efforts that aim to reform financing and reduce payments, the health sector should also provide leadership by strengthening supervision and standardizing treatment protocols for TB and MDR-TB, and also should monitor medical costs in collaboration with health insurance agencies, the major source of payments. Data from health-related information systems should be shared and used for evidence-based decision making.

Second, the health sector should engage in more communications about policies with the social welfare and security administration to improve the coordination of health insurance policies regarding the TB care delivery model. Because TB is a chronic communicable disease and a threat to public health, health insurance schemes should consider expanding coverage according to standard clinical protocols and reduce patient co-payments.

Lastly, the national TB program should develop better linkages with other social protection schemes to reduce direct medical and non-medical costs (transportation, food, basic living expenses, etc.) and indirect costs. Because TB is a disease of poverty, monitoring the coverage of TB services among the poorest 10% or 20% of the population will be likely to lead to significant improvements [5]. Integration of various social resources (medical assistance, poverty alleviation programs, charitable funds, etc.) is particularly important in low resource settings so that the most vulnerable and marginalized TB and MDR-TB patients receive sufficient social protections.

## Conclusions

The Chinese government is examining the mobilization and development of multi-source financing for the treatment of TB, in which social and government funds provide the greatest support. This approach, which is part of the movement towards UHC, requires increased accountability for the care of TB patients at multiple sectors and strong government leadership. All key stakeholders must take concrete actions before we can make significant progress in alleviating the financial burdens faced by TB and MDR-TB patients.

## Data Availability

Not applicable.

## Abbreviations

CDC: Center for Disease Prevention and Control
DRGs: Diagnosis Related Groups
DR-TB: Drug-resistant tuberculosis
GDP: Gross Domestic Product
MDR-TB: Multi-drug resistant tuberculosis
NCMS: New Cooperative Medical Scheme
SDGs: Sustainable Development Goals
TB: Tuberculosis
UEBMI: Urban Employee Basic Medical Insurance
URBMI: Urban Residence Basic Medical Insurance
URRBMI: Urban and Rural Residence Basic Medical Insurance
UHC: Universal Health Coverage
WHO: World Health Organization
